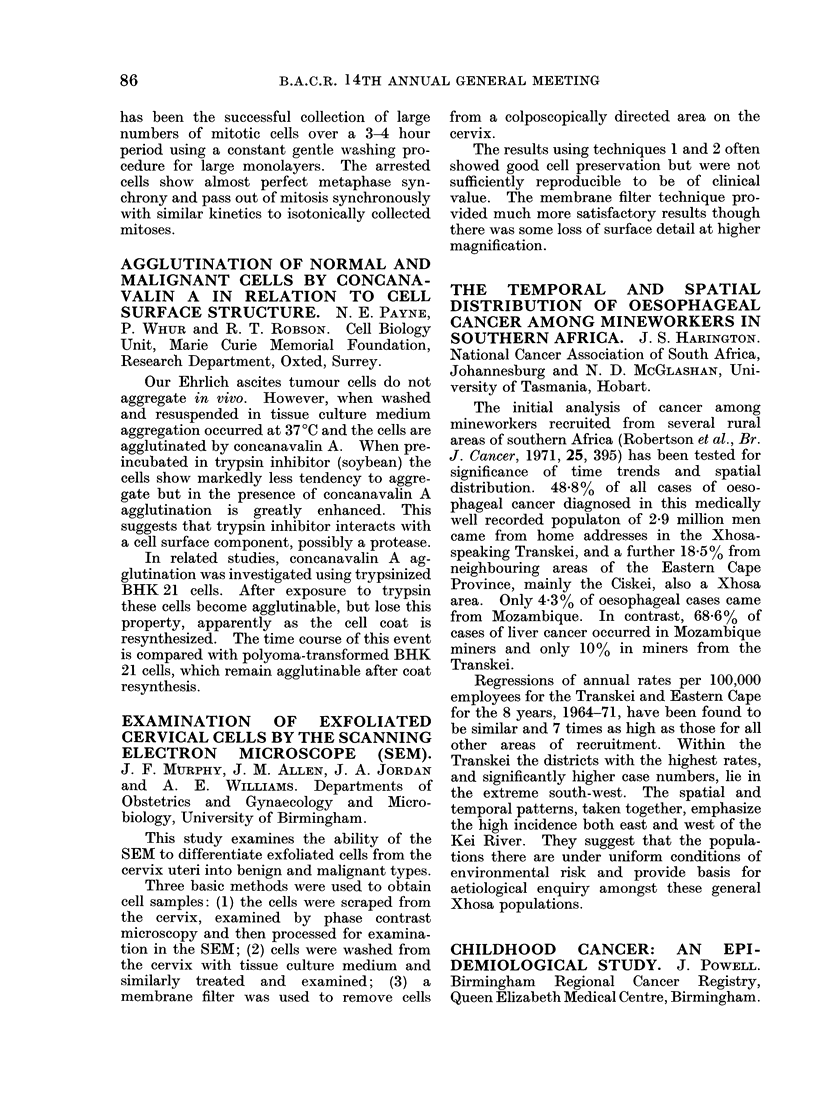# The temporal and spatial distribution of oesophageal cancer among mineworkers in Southern Africa.

**DOI:** 10.1038/bjc.1973.109

**Published:** 1973-07

**Authors:** J. S. Harington


### THE TEMPORAL AND SPATIAL DISTRIBUTION OF OESOPHAGEAL CANCER AMONG MINEWORKERS IN SOUTHERN AFRICA.

J. S. HARINGTON. National Cancer Association of South Africa, Johannesburg and N. D. MCGLASHAN, University of Tasmania, Hobart.

The initial analysis of cancer among mineworkers recruited from several rural areas of southern Africa (Robertson *et al.*, *Br. J. Cancer*, 1971, **25**, 395) has been tested for significance of time trends and spatial distribution. 48·8% of all cases of oesophageal cancer diagnosed in this medically well recorded populaton of 2·9 million men came from home addresses in the Xhosa-speaking Transkei, and a further 18·5% from neighbouring areas of the Eastern Cape Province, mainly the Ciskei, also a Xhosa area. Only 4·3% of oesophageal cases came from Mozambique. In contrast, 68·6% of cases of liver cancer occurred in Mozambique miners and only 10% in miners from the Transkei.

Regressions of annual rates per 100,000 employees for the Transkei and Eastern Cape for the 8 years, 1964–71, have been found to be similar and 7 times as high as those for all other areas of recruitment. Within the Transkei the districts with the highest rates, and significantly higher case numbers, lie in the extreme south-west. The spatial and temporal patterns, taken together, emphasize the high incidence both east and west of the Kei River. They suggest that the populations there are under uniform conditions of environmental risk and provide basis for aetiological enquiry amongst these general Xhosa populations.